# *Pc*Trim prevents early infection with white spot syndrome virus by inhibiting AP1-induced endocytosis

**DOI:** 10.1186/s12964-023-01059-7

**Published:** 2023-05-08

**Authors:** Xiao-Tong Cao, Lian-Jie Wu, Feng-Lin Xu, Xin-Cang Li, Jiang-Feng Lan

**Affiliations:** 1 Shandong Provincial Key Laboratory of Animal Biotechnology and Disease Control and Prevention, College of Animal Science and Veterinary Medicine, Shandong Agricultural University, Taian, 271018 China; 2 Key Laboratory of East China Sea Fishery Resources Exploitation, East China Sea Fisheries Research Institute, Chinese Academy of Fishery Sciences, Shanghai, 200090 China

**Keywords:** Tripartite motif (TRIM), AP1, White spot syndrome virus, Endocytosis, Dynamin, Immediate-early protein (IE1)

## Abstract

Viruses have evolved various strategies to achieve early infection by initiating transcription of their own early genes via host transcription factors, such as NF-κb, STAT, and AP1. How the host copes with this immune escape has been a topic of interest. Tripartite motif (TRIM) family proteins with RING-type domains have E3 ubiquitin ligase activity and are known as host restriction factors. Trim has been reported to be associated with phagocytosis and is also believed to be involved in the activation of autophagy. Preventing the virus from entering the host cell may be the most economical way for the host to resist virus infection. The role of TRIM in the early stage of virus infection in host cells remains to be further interpreted. In the current study, a crayfish TRIM with a RING-type domain, designated as *Pc*Trim, was significantly upregulated under white spot syndrome virus (WSSV) infection in the red swamp crayfish (*Procambarus clarkii*). Recombinant *Pc*Trim significantly inhibited WSSV replication in crayfish. RNAi targeting *Pc*Trim or blocking *Pc*Trim with an antibody promoted WSSV replication in crayfish. Pulldown and co-IP assays showed that *Pc*Trim can interact with the virus protein VP26. *Pc*Trim restricts the expression level of dynamin, which is involved in the regulation of phagocytosis, by inhibiting AP1 entry into the nucleus. AP1-RNAi effectively reduced the expression levels of dynamin and inhibited host cell endocytosis of WSSV in vivo. Our study demonstrated that *Pc*Trim might reduce early WSSV infection by binding to VP26 and then inhibiting AP1 activation, resulting in reduced endocytosis of WSSV in crayfish hemocytes.

**Video Abstract**

**Supplementary Information:**

The online version contains supplementary material available at 10.1186/s12964-023-01059-7.

## Introduction

Virus pathogenesis starts with the virus invading the host cells, where it then replicates and proliferates with the help of the energy and metabolic system of the host cell, which directly leads to cell structural damage and functional dysfunction. There are three main ways for viruses to enter host cells: membrane fusion and internalization into the cell, endocytosis into cells, and injection of the genome into cells. Endocytosis is the most common way for viruses to enter cells. Endocytosis mainly occurs through three pathways: clathrin- and dynamin-mediated endocytosis [1], caveolin-dependent endocytosis [2], and macropinocytosis [3]. WSSV enters cells by endocytosis, such as clathrin-mediated endocytosis, caveola-mediated endocytosis, and micropinocytosis [4–6]. The envelope protein VP24 of WSSV interacts with *Mj*plgR and is endocytosed into cells via clathrin [7]. Dynamin and clathrin are essential molecules in viral infection. After the virus infects an organism, it initiates the expression of its immediate-early protein. Viruses have evolved superior strategies to promote early infection and evade host immunity, while the host triggers an immune response to resist virus invasion. This is an invisible war and a competition.

WSSV, a virulent pathogen, is a circular double-stranded DNA virus that has a large genome (300 kb) and results in serious economic losses [8]. WSSV is been the most damaging viral disease in farmed crustaceans [9]. The current WSSV epidemic has resulted in high infection and mortality rates, infection in a wide range of crustaceans that is hard to control, and a certain threat to aquatic organisms. Currently, there are no effective prevention and control measures for WSSV. Thus, molecular insight into the mechanism that prevents viral infection is of fundamental importance. Although many studies have examined the immune defense against viral infection, our knowledge of the mechanism that prevents viral infection is still rudimentary.

Tripartite motif (TRIM) proteins, most of which contain a RING-type domain with a zinc finger motif, have E3 ubiquitin ligase activity [10]. TRIM proteins are increasingly known as crucial antiviral factors that inhibit the replication of multiple RNA viruses and DNA viruses. TRIM22 restrains hepatitis C virus (HCV) infection via the viral NS5A protein [11]. The SPRY domain of TRIM14 targets and degrades the viral NS5A protein in the immune defense against HCV infection [12]. In innate immunity, this is the first study in which TRIM5α was found to target multiple viruses, including retroviruses and lentiviruses, and prevent infection, except HIV infection in humans [13].

TRIM protects hosts from viral infection in a variety of ways. TRIM32 is involved in pathogen-infection resistance and the oxidative stress response in *Litopenaeus vannamei* [14]. TRIM5 can directly bind to virus proteins and then destroy the virus core and inhibits the replication of viruses by activating the NFκB signaling pathway [15]. Promyelocytic leukemia protein (PML), TRIM19, inhibits a wide range of RNA viruses and DNA viruses, such as herpesviruses, by organizing the nuclear bodies of PML [16, 17]. Host‒virus interactions are critical for successful replication and persistence within infected cells, viral spread between host cells and ultimately pathological outcomes. Therefore, understanding the interactions between viral and host proteins may provide crucial information for the development of new antiviral strategies. TRIM proteins suppress the replication of viruses by directly targeting viral proteins or regulating the innate immune responses that induce antiviral gene expression [18, 19].

Since virus pathogenesis starts with virus entry into cells, blocking virus entry into cells is a critical step for the host to resist viral infection; otherwise, stopping the virus will become more difficult. Half of TRIM proteins modulate autophagy, and a previous study showed that TRIM proteins can act both as autophagic cargo receptors and autophagy regulators [20]. Reports have shown that TRIM13 interacts with p62, inducing autophagy during ER stress [21]. TRIM2, a novel TRIM, plays an important role in antiviral immunity by limiting New World arenavirus (NWA) entry into host cells [22]. TRIM43 has been reported to inhibit herpesvirus infection by monitoring nuclear lamina integrity [23].

The present study details how the host responds to virus escape mechanisms. Virus invasion of host cells is accompanied by activation of the AP1 signaling pathway, which mediates expression of the endocytosis-related protein dynamin to achieve early infection in cells. The host inhibits the activation of the AP1 signaling pathway through *Pc*Trim, thereby limiting early viral transcription. Our study revealed that *Pc*Trim plays a critical role in resisting the endocytosis of WSSV in the early phase of viral infection.

## Materials and methods

### Sequencing and phylogenetic analysis of *Pc*Trim

The sequence of *Pc*Trim (XP_045581296) was amplified via PCR using primers and resequenced for confirmation. Similarity analysis was compared using blastx in the NCBI database (http://blast.ncbi.nlm.nih.gov). The domain architecture of *Pc*Trim was determined using SMART (http://smart.embl-heidelberg.de/).

### *Pc*Trim tissue distribution and expression profiles

Healthy crayfish (*P. clarkii*; 10–15 g each) were obtained from a fish market in Weishan, Shandong Province, China. The crayfish were kept for a week in tanks with aerated water at 22 °C before the infectious challenge. The *Pc*Trim mRNA tissue distributions and expression profiles were analyzed using qRT‒PCR with the primers *Pc*Trim-RT-F and *Pc*Trim-RT-R (Table 1). RNA samples from the hemocytes, heart, hepatopancreas, gills, stomach and intestine were evaluated. 18S rRNA was used as the reference with the primers 18SF and 18SR (Table 1). The *Pc*Trim expression profiles in hemocytes, the hepatopancreas and the stomach were measured via qRT‒PCR after *Staphylococcus aureus*, *Vibrio parahaemolyticus* or WSSV infection. The qRT‒PCR program was as follows: 94 °C for 2 min, followed by 40 cycles at 94 °C for 15 s and 60 °C for 30 s. The results were analyzed using the 2^−ΔΔCt^ method as previously described [24].

**Table 1:** Primers used in this study

Primers	Sequences (5’-3’)
*Pc*Trim-EX-F	TACTCAGGATCCATGTCTCTATATCCATCA
*Pc*Trim -EX-R	TACTCACTCGAGTTAAGAGCCATCGTTTTC
*Pc*Trim-RT-F	ACTCAATCTGCCAGGTCA
*Pc*Trim-RT-R	TGAAACATCTGGGAGGC
VP28-RT-F	AGCTCCAACACCTCCTCCTTCA
VP28-RT-R	TTACTCGGTCTCAGTGCCAGA
Dynamin-RT-F	GCTATCTATTGCTTGGTGGA
Dynamin-RT-R	ATCTAAGGTAAACGCTGGAA
IE1-RT-F	GCACAACAACAGACCCTACCC
IE1-RT-R	GAAATACGACATAGCACCTCCAC
18S-RT-F	TCTTCTTAGAGGGATTAGCGG
18S-RT-R	AAGGGGATTGAACGGGTTA
*Pc*Trim-Ri-F	GCGTAATACGACTCACTATAGGGCAAAGCCTTTTCCTCC
*Pc*Trim-Ri-R	GCGTAATACGACTCACTATAGGATCTGGTTGAGAATGACTG
AP1-Ri-F	GCGTAATACGACTCACTATAGGCGTGACAAGGCACCAGA
AP1-Ri-R	GCGTAATACGACTCACTATAGGCACCAGCCCAGGGATAT
Dynamin-Ri-F	GCGTAATACGACTCACTATAGGCAGTATGTCGGCAGTTAGGT
Dynamin-Ri-R	GCGTAATACGACTCACTATAGGGTCCGATAATGTGCAATGAT
GFP-RNAi-F	GCGTAATACGACTCACTATAGGTGGTCCCAATTCTCGTGGAAC
GFP-RNAi-R	GCGTAATACGACTCACTATAGGCTTGAAGTTGACTTGATGCC
Dynamin-ChIP-F	TATGGGTTGTGCATGGTCG
Dynamin-ChIP-R	AAACCCTGATTTCCAGCAAAC

### Recombinant protein purification and antibody preparation

*Pc*Trim was amplified using *Pc*Trim-Ex-F/R. The PCR procedure was as follows: 95 °C for 5 min, 40 cycles at 95 °C for 30 s, 58 °C for 35 s, 72 °C for 50 s, and one cycle at 72 °C for 10 min. Both the DNA fragment (*Pc*Trim) and the vector (pET-32a) were digested with the corresponding restriction enzymes at 37 °C for 10 min and placed into a water bath for 2 h at 22 °C with T4 ligase (Takara, China). Recombinant *Pc*Trim was expressed in *Escherichia coli* BL21 (DE3) cells and purified with His resin following the manufacturer’s instructions. A specific polyclonal antibody against *Pc*Trim was obtained by immunizing rabbits with purified *Pc*Trim.

### RNA interference (RNAi) assay

RNAi primers (*Pc*Trim-Ri-F/R and GFP-Ri-F/R), each linked to the T7 promoter, were used to amplify *Pc*Trim, AP1, dynamin and GFP as templates for dsRNA synthesis, following a previously reported method [25]. Crayfish were divided into two groups and injected with ds*Pc*Trim and dsGFP (20 μg). dsGFP was used as the reference. After 48 h of treatment, total RNA was extracted to evaluate RNAi efficacy via qRT‒PCR as mentioned above. A similar method was used for the knockdown of AP1 and dynamin.

WSSV infection assay. To analyze the role of *Pc*Trim in WSSV infection, crayfish were divided into two groups (3 crayfish in each group): Group 1 (control), crayfish were injected with His-Tag (30 μl, 1 μg/μl) and challenged with WSSV for 1 h; Group 2, crayfish were injected with His-*Pc*Trim (30 μl, 1 μg/μl) and challenged with WSSV for 1 h. At 60 h post challenge, genomic DNA was extracted from the gills of crayfish in the two groups using a genomic DNA extraction kit according to the manufacturer’s instructions (Vazyme, China). qRT‒PCR was used to quantify the number of WSSV copies in the crayfish using the VP28 primers VP28-RT-F/R (Table 1). Meanwhile, proteins were also extracted from the gills. Western blot analysis was performed using antiserum of VP28 as described previously [26].

To further study the role of *Pc*Trim in WSSV infection, ds*Pc*Trim or *Pc*Trim antiserum was injected into crayfish, and dsGFP or Actin antiserum was used as a control. Anti-*Pc*Trim and anti-actin antibodies were purified with protein A (Sangon Biotech, China) following the manufacturer’s instructions. The copy number of the WSSV genome and the level of major envelope proteins were analyzed according to the above method.

### Crayfish survival assay

To further confirm the role of Trim in resisting WSSV infection, 30 μl of His-*Pc*Trim was injected into crayfish (30 shrimp per group, 10–15 g each), who were then challenged with WSSV 1 h after the first injection. His-Tag was used as a control. The number of surviving crayfish was recorded every day in both groups. After RNAi of *Pc*Trim in vivo, the survival of crayfish infected with WSSV was also analyzed. Each crayfish was injected with 25 μg of ds*Pc*Trim or dsGFP, and then, 50 μl WSSV was injected after 48 h. The survival rates were determined according to the above method.

### Pull-down assay

A pull-down assay was performed to assess the interaction between WSSV and His-*Pc*Trim protein. Generally, purified His-Tag and His-*Pc*Trim (20 μg) were incubated with gill lysate (challenged with WSSV) in tubes with gentle rotation at 4 °C for 2 h. The pull-down assay was performed following a previously described method [25].

### Co-IP assay

To further analyze the in vivo interaction of *Pc*Trim with VP26, a Co-IP assay was performed using *Pc*Trim antiserum and VP26 antiserum. Protein A resin (20 μl; Sangon Biotech, China) was washed twice with 1 ml of PBS, and then, 1 μl of *Pc*Trim or VP26 antibodies was added with gentle rotation at 4 °C for 1 h. After the beads were washed three times with PBS, 1 ml of gill lysate was added and incubated in each tube at 4 °C for 2 h. The crayfish were injected with WSSV. After thorough washing with PBS, 25 μl PBS was added, and the samples were boiled with 2 × loading buffer for 5 min. The samples were analyzed via western blotting using *Pc*Trim and VP26 antibodies. A tube with pro-antiserum protein A resin was used as a control.

### Western blot analysis

Western blot was used to detect AP1 translocation. Tissue proteins were obtained from the intestine of normal crayfish and WSSV-challenged crayfish. Cytoplasmic proteins and nuclear proteins were extracted using a nuclear cytoplasmic protein extraction kit (BioTeke, China) following the instructions. The samples were separated via 12% SDS–polyacrylamide gel electrophoresis and then transferred onto nitrocellulose membranes using transfer buffer (25 mM Tris, 20 mM glycine, 0.037% SDS, 20% ethyl alcohol). The membranes were blocked for 2 h with nonfat milk (3%, in TBS 10 mM Tris-HCl, pH 7.5, 150 mM NaCl) and then incubated with antiserum against AP1 (1:10,000 dilution in blocking milk solution) for 4 h at room temperature. The membranes were washed three times with TBST (10 mM Tris-HCl, pH 7.5, 150 mM NaCl, 0.1% Tween-20) and TBS and incubated with HRP-conjugated goat anti-rabbit antibody at a 1:10,000 dilution in blocking milk solution (Frdbio, China). Finally, the membranes were washed three times with TBST and with TBS. ECL reagent was used to detect protein levels.

### Immunocytochemical analysis

To detect the subcellular localization of AP1 in the hemocytes of crayfish, immunocytochemical assays were performed following a previous report [27]. Hemocytes were collected from crayfish with anticoagulation, centrifuged at 600 × g for 5 min at 4 °C, and then washed two times with PBS. The hemocytes were deposited onto a glass slide with polylysine. The glass was incubated in 0.2% Triton X-100 for 5 min, washed with PBS, and blocked with 3% BSA for 30 min at 37 °C. Then, anti-AP1 antibody in 3% BSA was added and incubated overnight at 4 °C. The hemocytes were washed six times with PBS, incubated with rabbit secondary antibody conjugated with ALEXA 488 (1:1000 diluted in 3% BSA) for 2 h at 37℃, washed with PBS, incubated with DAPI, and washed again with PBS. Finally, the hemocytes were stained with Dil (Beyotime) and washed with PBS again. The slides were observed under an Andor fluorescence microscope.

### Fluorescence labeling and phagocytosis assay

To further confirm the role of *Pc*Trim, AP1 and dynamin in WSSV infection, phagocytic rates were examined. VP28 is the main envelope protein of WSSV, and FITC-labeled VP28 was used to simulate WSSV entry into cells. Recombinant GST-VP28 protein (400 μg) was added to 40 μl of glutathione resin, incubated at 4 °C for 2 h, washed three times with PBS, and centrifuged at 500 × g for 3 min to remove the supernatant. The cells were resuspended in FITC (0.001 mg/ml), incubated overnight at 4 °C in the dark, and washed six times with PBS. At 6 h after injection with His-*Pc*Trim or His-Tag, FITC-VP28 was injected. Crayfish hemocytes were collected and fixed with anticoagulant at 30 min postinjection and washed two times with PBS. Hemocytes were deposited onto a glass slide with polylysine, allowed to stand for 30 min, and then washed five times with PBS. Phagocytosis was observed and quantitated under a fluorescence microscope. The phagocytotic rate was defined as [hemocytes engulfing VP28/all hemocytes observed] × 100%. The phagocytosis assay was performed according to a previous report [28] and repeated three times.

Phagocytosis assays of anti-Trim, dsTrim, anti-AP1, dsAP1 and dsDynamin were performed according to the above method.

### Chromatin immunoprecipitation (ChIP)

ChIP was performed following a method described in a previous report [29, 30] using the primers dynamin-ChIP-F/R.

## Results

### The tissue distribution and expression profiles of *Pc*Trim

**Fig. 1:**
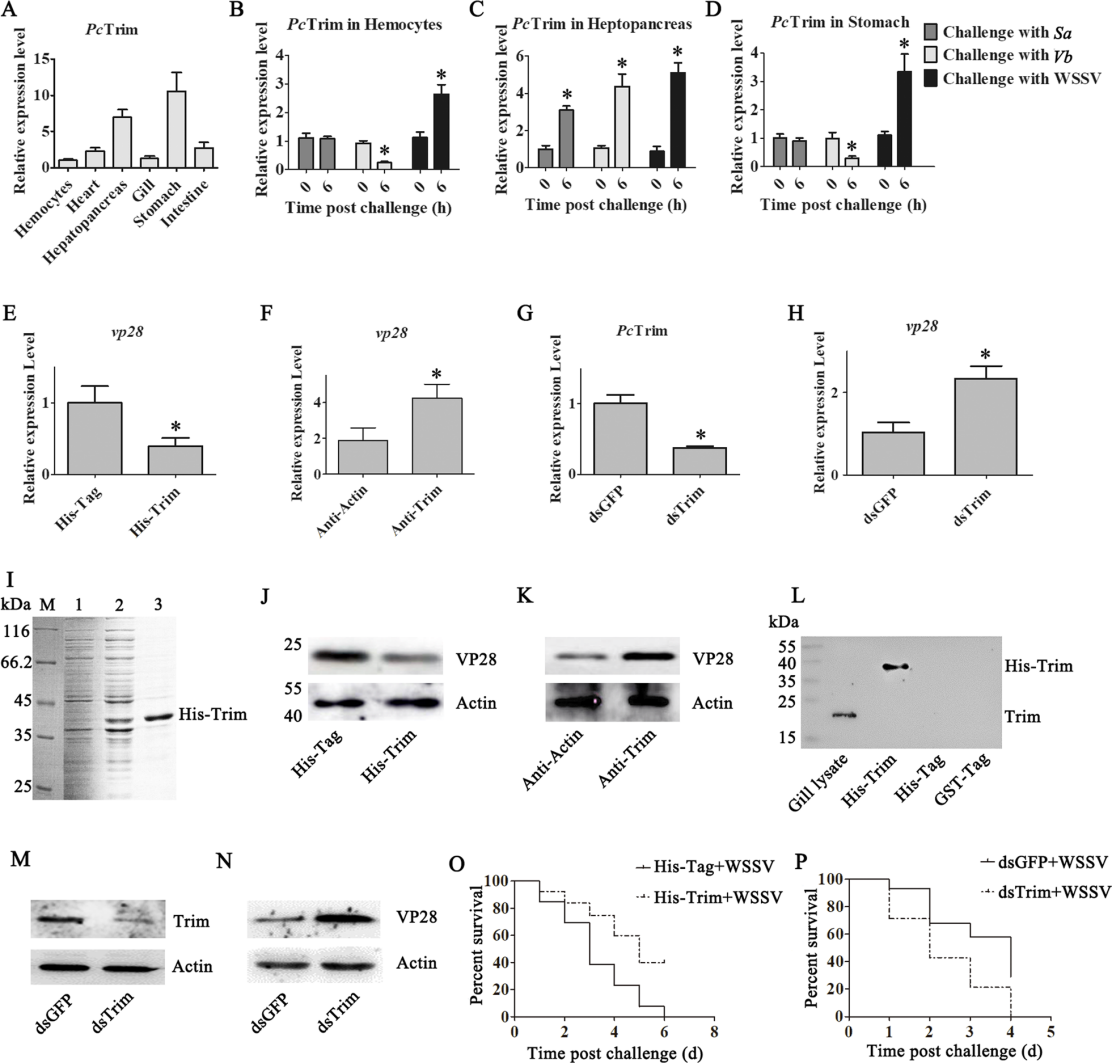
*Pc*Trim inhibits WSSV replication. **A** Tissue distribution of *Pc*Trim in crayfish at mRNA level. **B**–**D** Expression patterns of *Pc*Trim in hemocytes (**B**), hepatopancreas (**C**) and the stomach (**D**), as detected by qRT‒PCR. 18S rRNA was used as the control. qRT‒PCR and western blotting were used to analyze the amounts of WSSV (*vp28* was used as a marker of WSSV copy number). The expression levels of VP28 were analyzed in His-Trim injection crayfish (**E**, **J**), anti-Trim antibody injection crayfish (**F**, **K**) and dsTrim injection crayfish (H, N), and His-Tag, anti-Actin antibody and dsGFP were used as controls. The expression level of *Pc*Trim was measured by qRT‒PCR (**G**) and western blotting (**M**) 48 h after dsTrim injection. **I** Recombinant expression and purification of *Pc*Trim in *E.coli*. Lane 1, total proteins from *E.coli* with *Pc*Trim-pET-32a, without ITPG induction; Lane 2, total proteins from *E.coli* with *Pc*Trim-pET-32a, with ITPG induction; Lane 3, purified recombinant *Pc*Trim. **L** *Pc*Trim was detected using the *Pc*Trim polyclonal antibodies. Crayfish were divided into two groups: the groups were injected with either His-Tag or His-Trim, and then, all the crayfish were injected with WSSV (**O**); two groups of crayfish were injected with either dsGFP or dsTrim, and then, the crayfish were challenged with WSSV after *Pc*Trim-RNAi treatment (**P**). The survival rate of crayfish was calculated. The asterisk indicates a significant difference, *p* < 0.05

The tissue distribution of *Pc*Trim in hemocytes and the heart, hepatopancreas, gills, stomach and intestine was analyzed via qRT‒PCR. The results suggest that *Pc*Trim is distributed in all the tested tissues, with higher expression levels in the hepatopancreas and stomach and lower expression levels in hemocytes, the heart, the gills and the intestine (Fig. 1A). Hemocytes play an important role in innate immunity. Therefore, the expression profiles of *Pc*Trim in hemocytes, the hepatopancreas and the stomach were analyzed to explore whether *Pc*Trim expression responds to bacterial and virus challenges. The results showed that the expression level of *Pc*Trim was upregulated in hemocytes, the hepatopancreas and the stomach at 6 h after challenge with WSSV; in the hepatopancreas, the expression of *Pc*Trim was upregulated after challenge with *S. aureus* and *Vibrio*; and in hemocytes and the stomach, it was downregulated after challenge with *Vibrio* and did not change after challenge with *S. aureus* (Fig. 1B–D).

### PcTrim inhibit WSSV repliction

To determine whether *Pc*Trim has antiviral properties, His-*Pc*Trim (Fig. 1I) was injected into crayfish, and the amount of WSSV in the gills was measured via qRT‒PCR (Fig. 1E) and western blot (Fig. 1J) at 60 h after WSSV challenge. The results showed that His-*Pc*Trim reduced the copies of WSSV. The purity of *Pc*Trim antibody was detected (Fig. 1L). To confirm these results, anti-*Pc*Trim was injected into crayfish, and the amount of WSSV was detected via qRT‒PCR (Fig. 1F) and western blot (Fig. 1K) after WSSV challenge. The expression of *Pc*Trim was successfully altered after dsTrim injection at both the transcriptional level (Fig. 1G) and the ptrotein level (Fig. 1M). The WSSV replication levels in ds*Pc*Trim injection or anti-*Pc*Trim injection crayfish were significantly higher than those in the other crayfish (Fig. 1F, K, H, N).

To further investigate the role of *Pc*Trim in vivo, the survival rate was detected after crayfish were injected with His-*Pc*Trim (with WSSV challenge 2 h later) and ds*Pc*Trim (with WSSV infection after *Pc*Trim-RNAi treatment). The survival rate of the His-*Pc*Trim-injected crayfish was significantly higher than that of His-Tag-WSSV-injected crayfish (Fig. 1O). The survival rate of the ds*Pc*Trim-WSSV-injected crayfish was significantly lower than that of dsGFP-injected crayfish (Fig. 1P). These data suggest that *Pc*Trim significantly increases the survival rate of WSSV-challenged crayfish.

### *Pc*Trim binds to WSSV by interacting with VP26

**Fig. 2:**
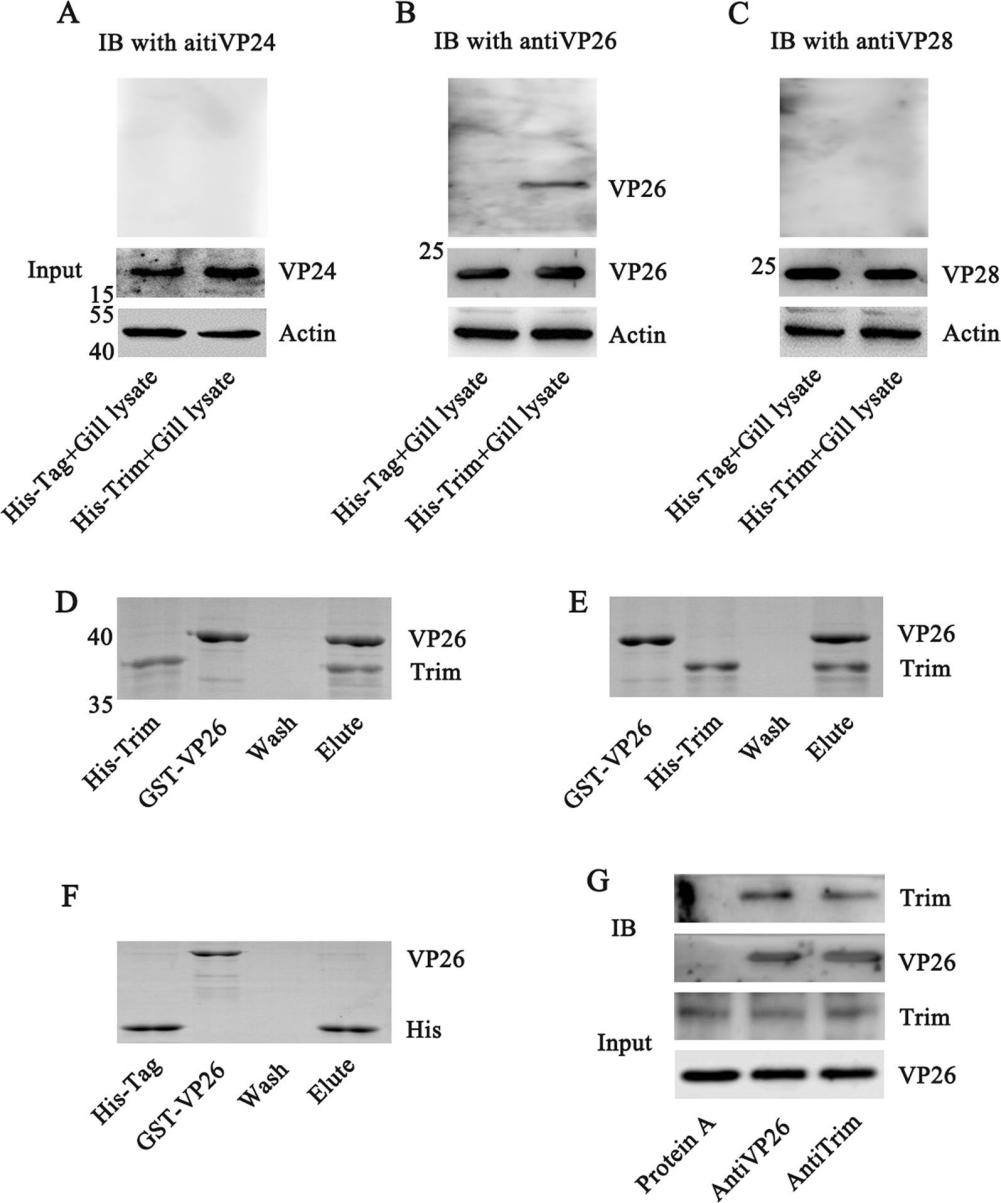
*Pc*Trim interacts with VP26. Far western blot was used to screen the interaction between VPs and Trim. Lysates of gills were incubated with His resin containing His-Trim (**A**, **B**, **C**), and then, three antibodies (anti-VP24, anti-VP26, and anti-VP28) were used to detect the presence of VP24, VP26 and VP28. His-Tag was used as negative control. The β-Actin was used as inner protein control. VP24, VP26 and VP28 were detected in each samples. Only VP26 was detected to interact with His-Trim (**B**). A pull-down assay was used to analyze the interaction between Trim and VP26. His-Trim was first bound to His-resin, and then, the resin was incubated with GST-VP26. After 3 washes with PBS, protein residues were analyzed via SDS‒PAGE (**D**). The interaction between GST-VP26 and His-Trim (**E**) or between GST-VP26 and His-Tag (**F**) was analyzed using the above methods with GST resin or His resin. The co-IP assay was performed using anti-*Pc*Trim and anti-VP26 antibodies and gill lysates from WSSV-infected crayfish. A tube with antiserum-free protein A resin was used as a control (**G**)

The above results revealed that *Pc*Trim inhibits WSSV replication, but the mechanism remains unclear. The interaction between *Pc*Trim and WSSV was detected. We determined whether the recombinant protein binds to the native WSSV proteins using a pull-down assay. The results showed that *Pc*Trim interacted with the viral protein VP26 (Fig. 2B) but not with VP24 (Fig. 2A) or VP28 (Fig. 2C).

In further investigation of the interaction of *Pc*Trim and VP26, pulldown assay results suggested that His-*Pc*Trim interacted with GST-VP26 (Fig. 2D, E). A co-IP assay was also performed using gills from WSSV-infected crayfish to detect the interaction of *Pc*Trim and VP26. The results revealed that native *Pc*Trim and native VP26 in gills interact with each other (Fig. 2G).

### *Pc*Trim inhibits the endocytosis of WSSV in hemocytes

**Fig. 3:**
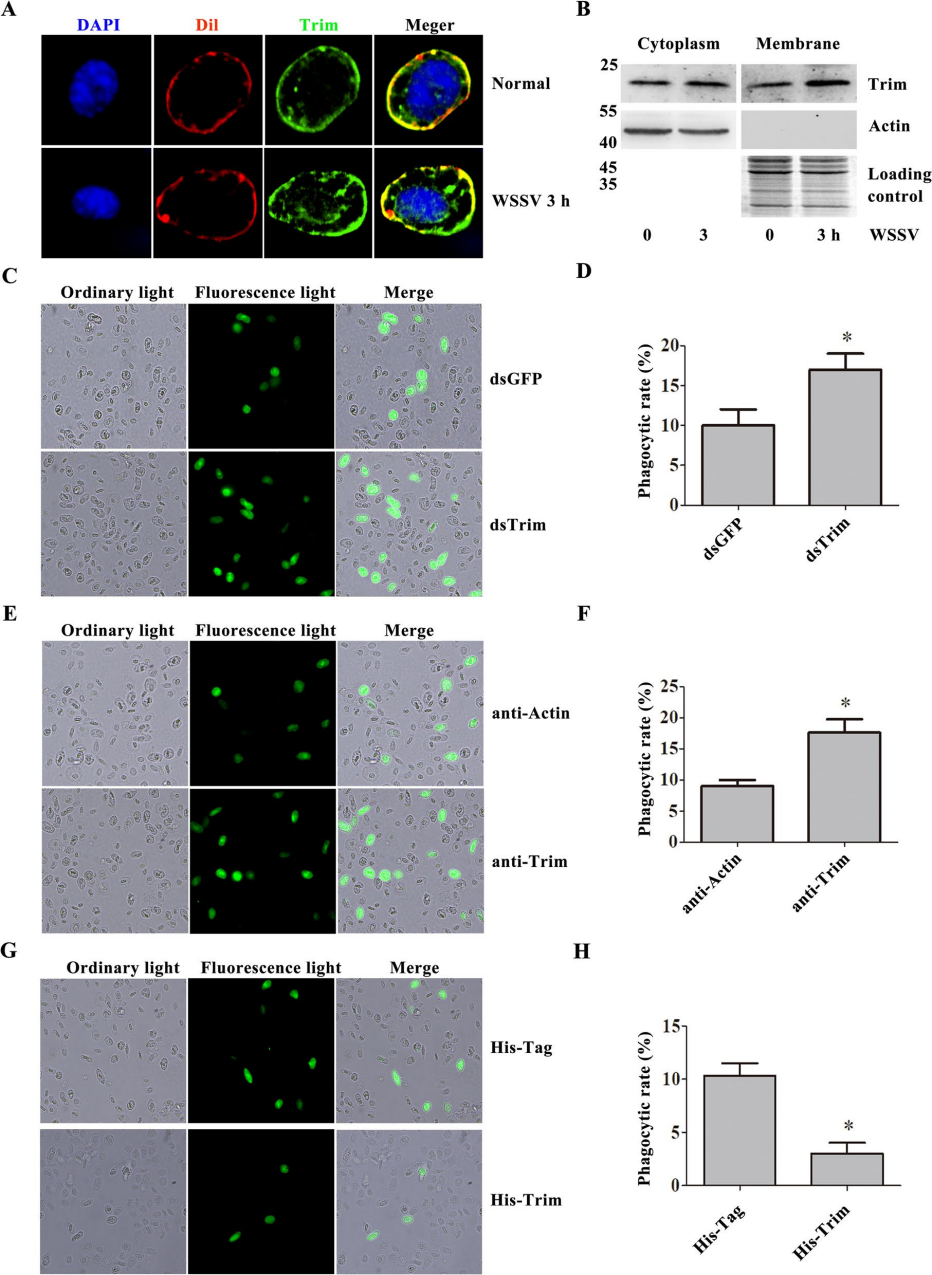
*Pc*Trim inhibits WSSV endocytosis in crayfish. The subcellular localization of *Pc*Trim was detected using an immunocytochemical assay (**A**) and western blot (**B**). Dil was used to label the cell membrane, and DAPI was used to label the cell nuclei. The role of *Pc*Trim in phagocytosis was studied by analyzing the phagocytosis rate of FITC-labeled VP28. After RNAi of Trim, FITC-labeled VP28 was injected into crayfish. Hemocytes were collected 30 min after VP28 injection (**C**), and phagocytosis of VP28 by hemocytes was observed via microscopy. **D** The phagocytosis rate after Trim-RNAi treatment was calculated using the described formula. dsGFP was used as a control. **E** After blocking Trim via injection with anti-Trim antibody, the rate of VP28 phagocytosis by hemocytes was analyzed with a fluorescence microscope. **F** The phagocytosis rate after Trim blocked. Anti-actin was used as a control. **G** Hemocyte phagocytosis observed under a fluorescence microscope after rTrim injection. **H** The phagocytosis rate after His-Trim injection. The asterisk indicates a significant difference, *p* < 0.05

The above study demonstrated that *Pc*Trim can bind with VP26. VP26 is one of the major outer membrane proteins of WSSV and is closely related to early WSSV infection. We detected the subcellular location of *Pc*Trim, which is mainly located on the membrane and in the cytoplasm (Fig. 3A, B), and found that it was unregulated after WSSV challenge (Fig. 3A, B). This result suggests that *Pc*Trim may be involved in the endocytosis of WSSV. FITC-labeled VP28 was injected after ds*Pc*Trim, anti-Trim or His-Trim injection to detect the phagocytic rate of hemocytes in crayfish. The results showed that the phagocytic rate of hemocytes was increased significantly in *Pc*Trim-RNAi (Fig. 3C, D) crayfish and anti-Trim injection (Fig. 3E, F) crayfish and was decreased in His-Trim injection (Fig. 3G, H) crayfish. These results suggest that *Pc*Trim inhibits the endocytosis of WSSV in crayfish.

### *Pc*Trim induces AP1 translocation into the nucleus

**Fig. 4:**
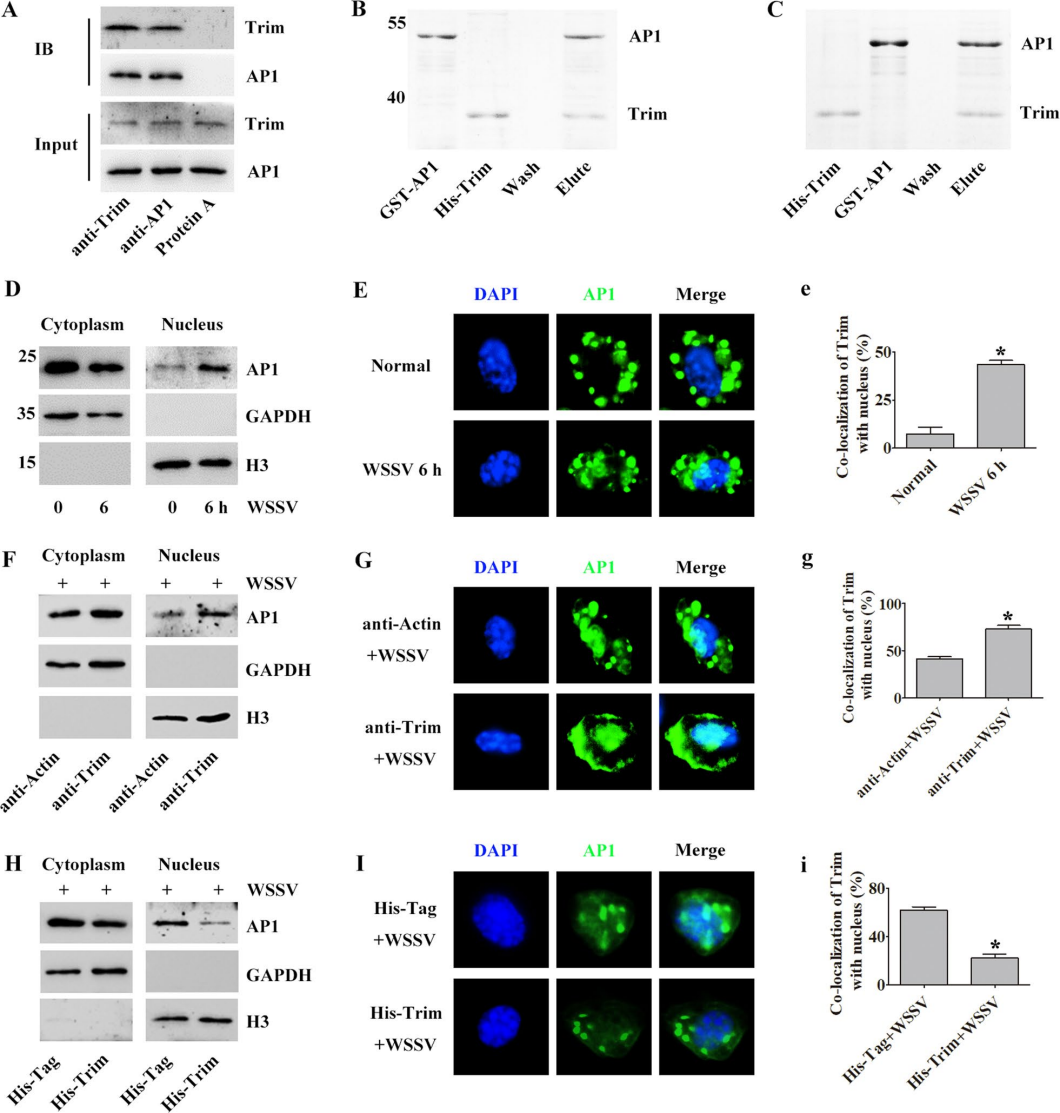
*Pc*Trim inhibits AP1 translocation from the cytoplasm into the nucleus. CoIP (**A**) and pull-down (**B**, **C**) assays were used to analyze the interaction between Trim and AP1. **D** The subcellular localization of AP1 was detected by western blot in intestine after WSSV challenge. GAPDH and H3 were used as controls for the cytoplasmic or nuclear proteins, respectively. AP1 translocation into the nucleus in hemocytes was detected in WSSV-challenged crayfish using an immunocytochemical assay. Normal crayfish were used as a negative control. The amount of AP1 localization in the nucleus was determined (**E**). The subcellular distribution of AP1 was detected by western blotting after injection of anti-Trim (**F**) or His-Trim (**H**) antibody in crayfish. Anti-Actin antibody and His-Tag were used as controls. **G**, **I** AP1 translocation into the nucleus in hemocytes was detected with an immunocytochemical assay after anti-Trim antibody (**G**) or His-Trim (**I**) injection. Anti-Actin antibody and His-Tag were used as controls. The distribution of AP1 in the nucleus was determined (**e**, **g**, **i**). The asterisk indicates a significant difference, *p* < 0.05

The above experiments demonstrate that *Pc*Trim inhibits the endocytosis of WSSV. AP1 plays a very important role in early virus infection. Therefore, we examined the potential role of *Pc*Trim in the activation of the AP1 signaling pathway by assessing the translocation of AP1 into the nucleus. CoIP and pull-down assays showed that Trim can interact with AP1 (Fig. 4A–C). AP1 translocated from the cytoplasm into the nucleus after WSSV infection, as shown by WB and IC assays (Fig. 4D–e). When Trim was blocked with anti-Trim antibody, more AP1 translocation into the nucleus was observed than in the anti-Actin injection group (Fig. 4F–g). However, in *Pc*Trim-injected crayfish, less AP1 translocation into the nucleus was observed than in His-Tag-injected crayfish (Fig. 4H–i). These results suggest that *Pc*Trim reduced phagocytosis of WSSV by host cells by inhibiting the activity of AP1.

### AP1 mediates the endocytosis of WSSV

**Fig. 5:**
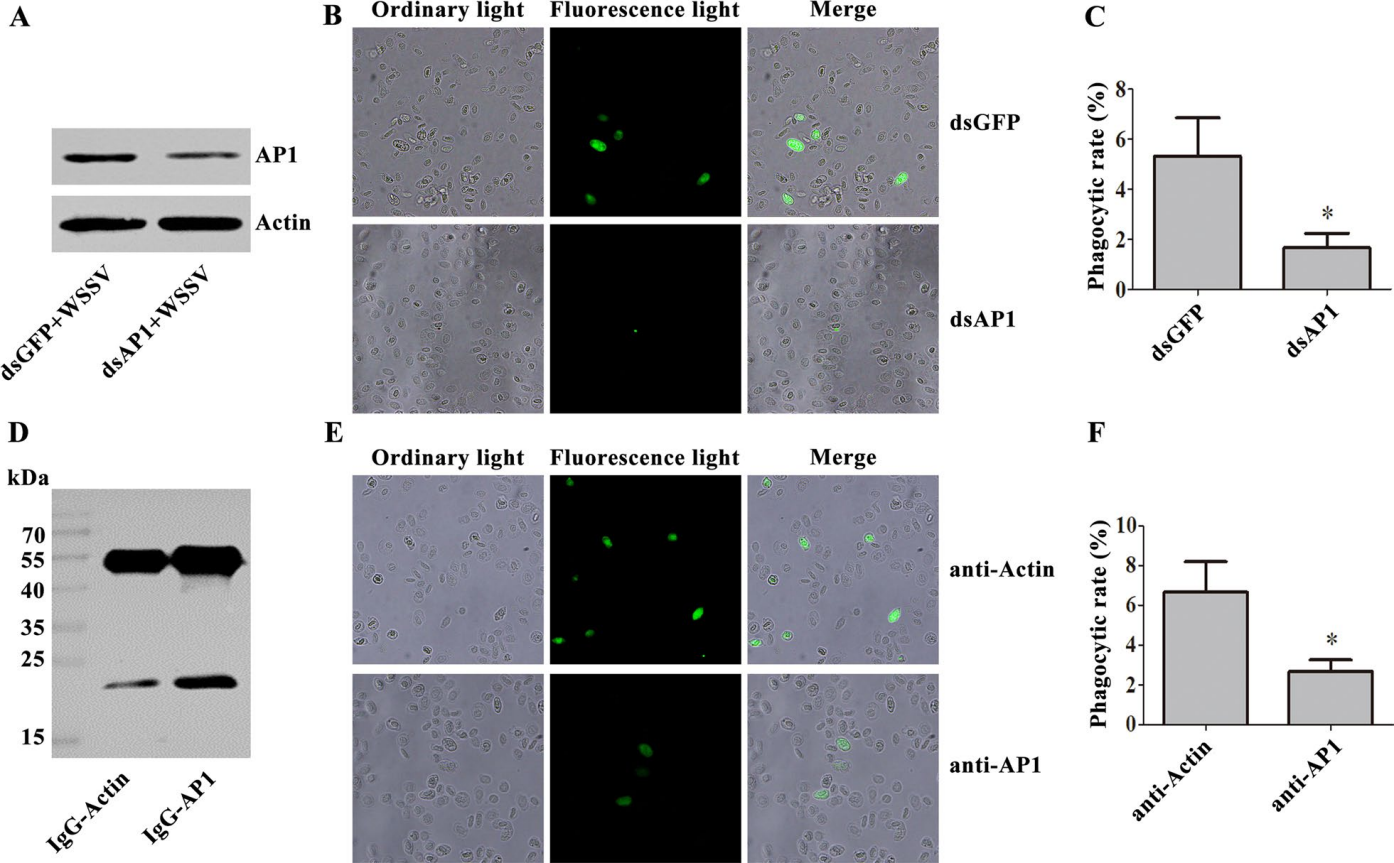
AP1 mediates WSSV endocytosis in crayfish. **A** The efficiency of AP1-RNAi was detected by western blot. **B** Hemocyte phagocytosis was observed under a fluorescence microscope after AP1-RNAi. **C** The phagocytosis rate of VP28 was studied after AP1-RNAi. **D** The purified antibody against AP1 was analyzed by SDS‒PAGE. **E** Hemocyte phagocytosis observed under a fluorescence microscope after blocking with anti-AP1 antibody. **F** The phagocytosis rate of VP28 after AP1 blockade. The asterisk indicates a significant difference, *p* < 0.05

After AP1-RNAi (Fig. 5A) and blocking AP1 with purified anti-AP1 antibody (Fig. 5D), the phagocytic rate of hemocytes was detected 30 min after FITC-labeled VP28 injection. The results showed that the phagocytic rate in dsAP1-injected crayfish (Fig. 5B, C) or anti-AP1-injected crayfish (Fig. 5E, F) was significantly decreased compared with that in the control crayfish. These results indicate that AP1 mediates the endocytosis of WSSV.

### *Pc*Trim decreases the expression of phagocytosis-related genes by inhibiting AP1 activity

**Fig. 6:**
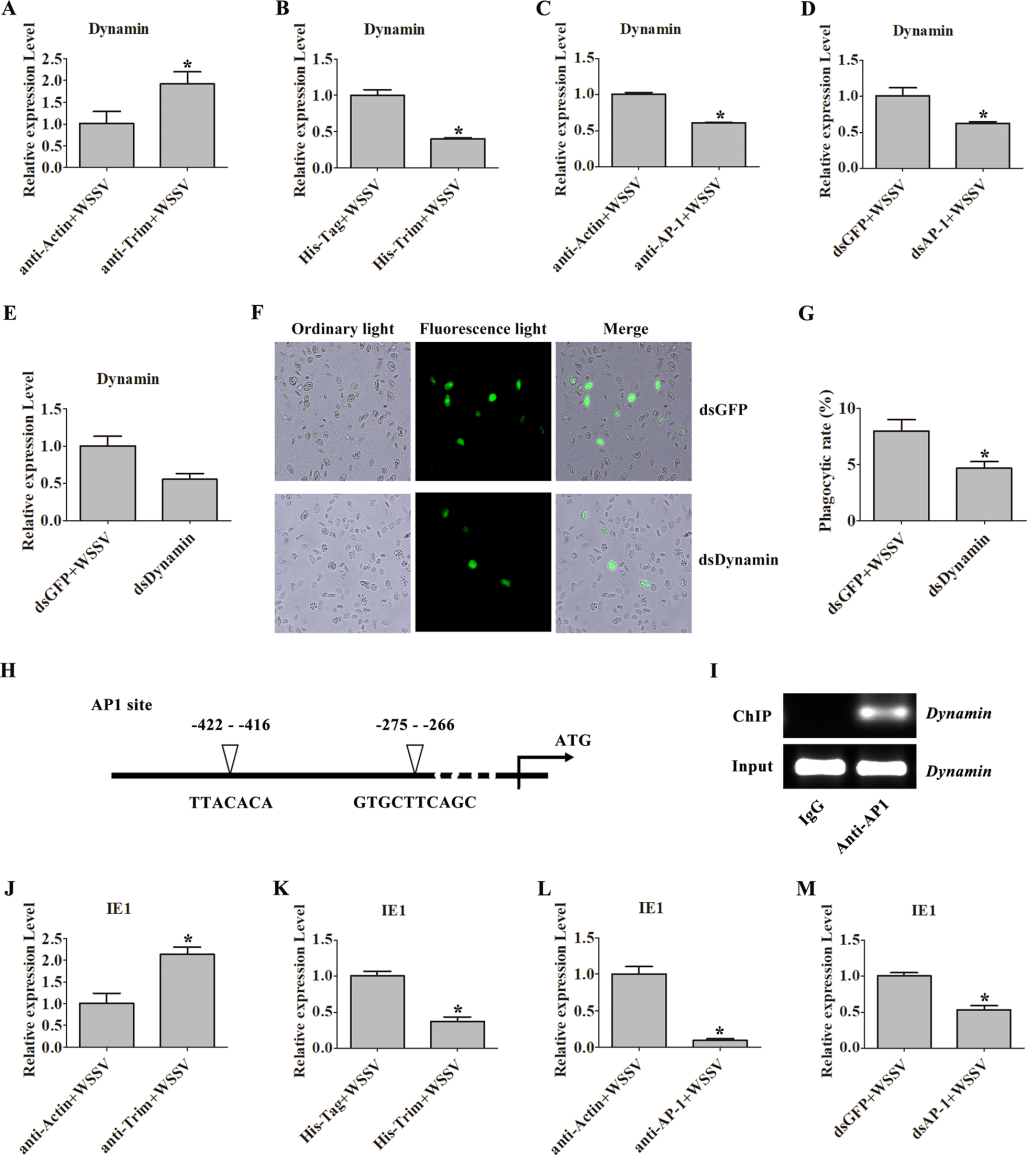
*Pc*Trim decreases dynamin expression by inhibiting the activity of AP1. **A–D** The expression of *dynamin* in crayfish injected with anti-Trim antibody (**A**), His-Trim (**B**), anti-AP1 antibody (**C**) or dsAP1 (**D**) was analyzed via qRT‒PCR. Anti-Actin antibody, His-Tag and dsGFP were used as controls. **E** The *dynamin* RNAi efficiency was analyzed via qRT‒PCR. **F** Hemocyte phagocytosis was observed under a fluorescence microscope after *dynamin* RNAi treatment. **G** The phagocytosis rate of VP28 after *dynamin* RNAi treatment. (H) The 5’ untranslated region of dynamin was analyzed using the online Promoter Scan tool. Two AP1 binding sites were found in the genomic regulatory region of dynamin. **I** A ChIP assay was used to detect the AP1 binding sites in the dynamin promoter. **J**–**M** The expression level of *IE1* in crayfish injected with anti-Trim antibody **J**, His-Trim **K**, anti-AP1 antibody **L** or dsAP1 **M** was analyzed via qRT‒PCR. The asterisk indicates a significant difference, *p* < 0.05

The GTPase dynamin is essential for endocytosis and controls the formation of constricted coated pits [31]. ASFV enters cells via clathrin-mediated endocytosis, which is dependent on dynamin GTPase activity [32]. To further investigate the mechanism by which *Pc*Trim mediates the activation of AP1 to inhibit the early WSSV infection, we analyzed the expression of dynamin. In anti-Trim injection crayfish, the expression of dynamin was increased after WSSV challenge compared with expression in anti-Actin injection crayfish (Fig. 6A). In the His-Trim injection crayfish, the expression level of dynamin was decreased after WSSV challenge compared with expression in the His-Tag injection crayfish (Fig. 6B). When AP1 was blocked via injection with anti-AP1 antibody or by injection with dsAP1 for AP1-RNAi, the expression level of dynamin was decreased after WSSV challenge compared with anti-Actin or dsGFP injection in crayfish (Fig. 6C, D). After dynamin-RNAi (Fig. 6E), the phagocytic rate was decreased compared with that in the dsGFP-injected crayfish (Fig. 6F, G).

The above results suggest that *Pc*Trim is upstream of AP1 and regulates the expression of dynamin by regulating the activity of AP1 to suppress WSSV endocytosis. However, whether dynamin expression is directly or indirectly regulated by AP1 remains uncertain. To identify putative AP1-binding sites in the promoter sequence of the dynamin of interest, the promoter region of dynamin was cloned, and two AP1 sites were identified in the promoter sequence (Fig. 6H). Next, we conducted a ChIP assay with anti-AP1 antibody, analyzed the DNA fragment obtained, amplified the dynamin sequence of interest via RT‒PCR (Fig. 6I), and a positive signal was detected. This suggests that AP1 can directly transcriptionally regulate dynamin expression. Once the virus enters the host cell, it launches its own early replication system and initiates the expression of the immediate-early protein. We further analyzed the expression level of WSSV IE1. In crayfish in which Trim was blocked with anti-Trim antibody, the expression level of IE1 was increased (Fig. 6J). In His-Trim injection crayfish, the expression level of IE1 was decreased (Fig. 6K) compared with crayfish with His-Tag injection. When AP1 was blocked with anti-AP1 antibody or AP1-RNAi was induced with dsAP1, the expression level of IE1 was decreased (Fig. 6L, M) compared with that in crayfish injected with anti-Actin antibody or dsGFP. These results suggest that *Pc*Trim can regulate dynamin expression by inhibiting the activity of AP1 to suppress endocytosis, thereby restraining early WSSV infection.

## Discussion

Viral entry into host cells is a critical step toward successful infection, which is a major determinant of pathogenesis. Therefore, preventing viruses from entering cells is the key step to inhibit viral infection. In the current study, we expounded on the mechanism by which *Pc*Trim prevents WSSV from entering host cells by restraining the endocytosis of WSSV virions to inhibit early infection.

Both virus infection of host cells through binding to host proteins and host defense against virus infection usually require binding to viral proteins. Therefore, it is very important to screen viral proteins that interact with target proteins. To ascertain whether *Pc*Trim binds to viral proteins, we screened several major structural envelope proteins of WSSV and ultimately found that *Pc*Trim interacts with VP26. The C-type lectin *Mj*svCL presents virions to the cell-surface receptor calreticulin to promote viral infection by interacting with the viral protein VP28 [33]. *Pc*PHB1 interacts with the structural envelope proteins VP24, VP26 and VP28 of WSSV and prevents viral infection in the red swamp crayfish *Procambarus clarkii* [25]. The structural protein VP26 is one of the major outer membrane proteins of WSSV and is closely related to early viral infection. Therefore, the suppression mechanism may not be that simple.

As a WSSV pattern recognition receptor, *Mj*SRC promotes phagocytosis of the virus, ultimately resulting in degradation of WSSV in lysosomes [6]. To answer these questions, we knocked down the expression of *Pc*Trim in crayfish, and the number of WSSV virions entering the cells was determined. The results showed that the number of WSSV virions that entered cells was increased after *Pc*Trim-RNAi treatment compared with the control group (Fig. 3C). This result was also verified by injection of *Pc*Trim antiserum (Fig. 3E) and *Pc*Trim protein (Fig. 3G) in crayfish. However, further questions remain to be approached.

**Fig. 7:**
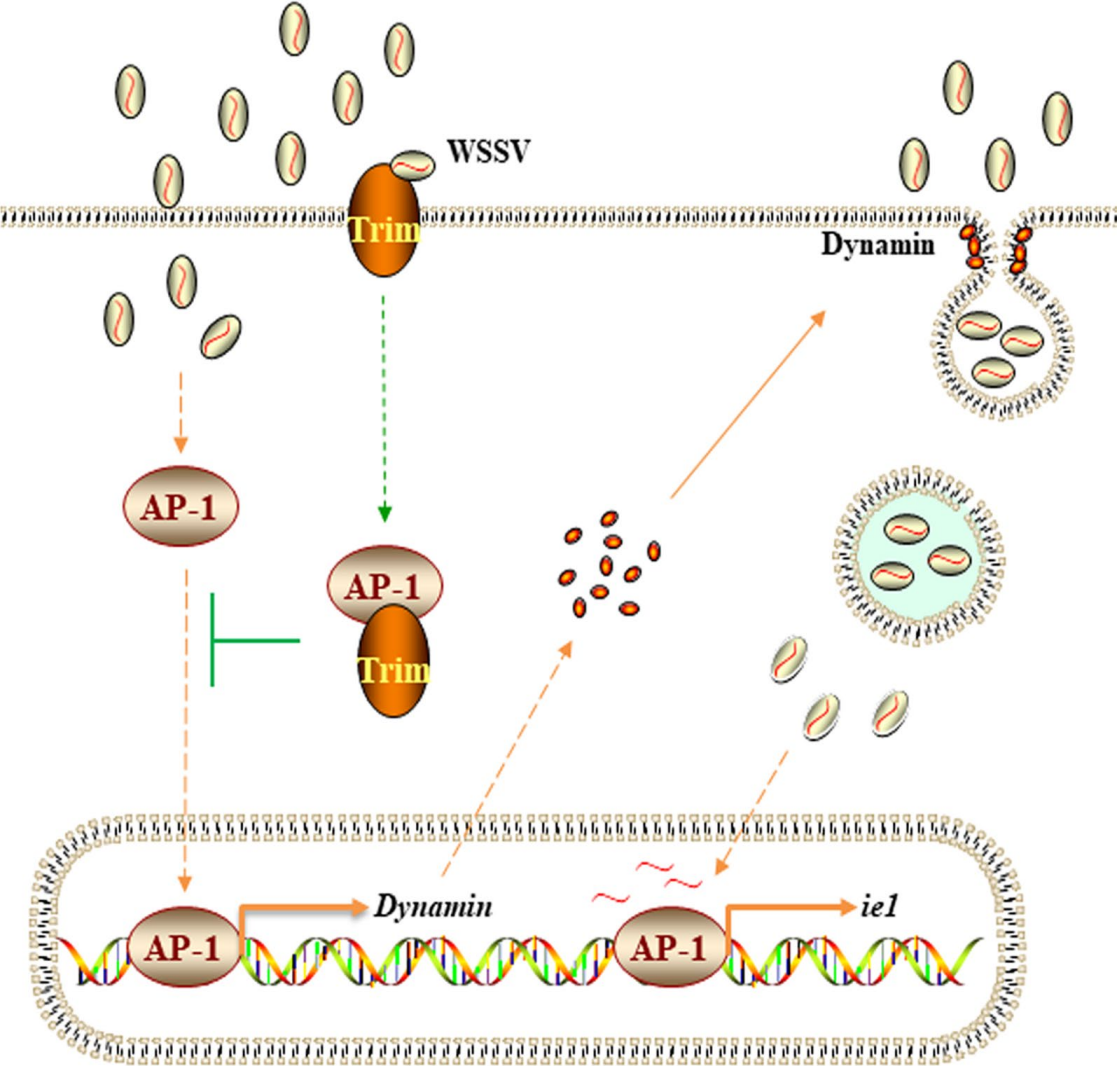
Model of the *Pc*Trim-mediated antiviral mechanism. In the early stage of WSSV infection in crayfish cells, Trim, which localizes to the membrane, recognizes the WSSV protein VP26 and binds AP1 to inhibit AP1 entry into the nucleus. Activated AP1 enhances the expression of the phagocytosis-related protein dynamin, a host protein on which WSSV invasion depends, thereby promoting the early replication of WSSV

Viruses have evolved superior strategies to avoid host immunity. It has been reported that endocytosed WSSV virions are directed to the endosomal delivery system via *Cq*VCP, thereby avoiding autophagic degradation, delivering the viral genome into the nucleus, and facilitating viral infection [34]. *Mj*pIgR has been reported as a WSSV receptor that can interact with the WSSV protein VP24 extracellularly and helps WSSV enter host cells through the pIgR-CaM-clathrin endocytic pathway [7]. Virus entry into cells is accompanied by the activation of certain signaling pathways, such as the NF-κB, JAK/STAT and AP1 signaling pathways [35–37]. The zinc finger protein ZNF394 inhibits the transcriptional activities of AP1 [38]. It has also been reported that TRIM5 activates AP1 and restricts the transduction of HIV-1 [39]. The regulation of AP1 activity by Trim remains controversial. The AP1 transcription factor Fosl-2 regulates autophagocytosis and controls the differentiation of cardiac fibroblasts [40]. AP1 can bind to the promoter of the CMV major early gene to initiate viral replication [41]. There have been many reports that viruses enhance early replication by activating several signaling pathways, but in this case, the mechanism of host defense against virus infection has remained elusive. Our results showed that after AP1-RNAi and injection of anti-AP1 antiserum, WSSV entry into cells was decreased compared with that in the control group (Fig. 5). AP1 may promote early infection by facilitating the endocytosis of WSSV in crayfish. Transcription factors can regulate gene expression and perform corresponding functions when they enter the nucleus. *Pc*Trim inhibits entry of the transcription factor AP1 into the nucleus by binding to AP1 (Fig. 4), and we were surprised to find *Pc*Trim distributed on the cell membrane, and the amount of *Pc*Trim increased after WSSV infection. Dynamin is essential for endocytosis. When Trim blocked AP1 translocation into the nucleus from the cytoplasm, the expression level of dynamin was decreased (Fig. 6), which led to the active endocytosis of WSSV by host cells (Fig. 6). These results suggest that when WSSV infects the crayfish, *Pc*Trim located on the cell membrane may recognize the WSSV by interacted with VP26 and transmits the signal into the cell, then *Pc*Trim interacts with AP1 to inhibit AP1 entering the nucleus, thus inhibiting the early endocytosis of WSSV. Interestingly, we found that *Pc*Trim was localized not only on cell membrane, but also on the nuclear membrane. Trim which located on the nuclear membrane may inhibit AP1 transfer into the nucleus from cytoplasm by blocking AP1. WSSV was found to synthesize proteins in the cytoplasm and complete assembly in the nucleus [34]. In the other hand it may inhibit the assembly of WSSV by combining with VP26, thus inhibiting the replication of WSSV, so as to achieve a dual protective effect (Fig. 7).

In conclusion, virus infection of host cells requires the phagocytosis activity of host cells. *Pc*Trim reduces the amount of WSSV entering host cells by inhibiting AP1 entry into the nucleus, thereby restraining the AP1-mediated phagocytosis-associated expression level of dynamin, which prevents the virus from invading host cells.

## Data Availability

The datasets used for the current study are available from the corresponding author on reasonable request.
